# Scanning Electron Microscopy Analysis of Smear Layer Removal Ability of Conventional Endodontic Irrigation Regimen, MTAD, and QMix™ Versus a Mixture of Azadirachta indica and Citrus limon: An In Vitro Study

**DOI:** 10.7759/cureus.42877

**Published:** 2023-08-02

**Authors:** Nagammai Meyappan, Mageshwari Mahadevan, Niranjana Devi Manimaran, Benin Paulaian, Rajesh Gopal, Naveen Kumar

**Affiliations:** 1 Department of Conservative Dentistry and Endodontics, Rajas Dental College and Hospital, Tirunelveli, IND; 2 Department of Conservative Dentistry and Endodontics, Private Practice, Thiruvananthapuram, IND

**Keywords:** intra-radicular smear layer, root canal system, removal of the smear layer, 3%naocl + 17%edta + 2%chx, neem extracts, citrus limon, azadirachta indica, mtad, scanning electron microscopy

## Abstract

Introduction: Smear layer removal from root canals aid in the penetration of both irrigants and endodontic sealer into the dentinal tubules, thereby improving the efficacy of endodontic treatment. The aim of this in vitro study was to compare the smear layer removal ability of a conventional endodontic irrigation regimen, MTAD (mixture of tetracycline, acid, and detergent), and QMix™ (Dentsply Sirona, Charlotte, North Carolina, United States) with that of a mixture of herbal irrigants, namely, aqueous extracts of *Azadirachta* *indica* (neem) and *Citrus limon *(lemon)*,* evaluated using scanning electron microscopy (SEM).

Materials and methods: We selected 40 extracted human premolar teeth for the study, which we randomly divided into five groups (eight samples each) according to irrigation solution: (i) Group A (normal saline); (ii) Group B (conventional endodontic regimen, 3% sodium hypochlorite (NaOCl) + 17% ethylenediamine tetraacetic acid (EDTA) + 2% chlorhexidine (CHX)); (iii) Group C (MTAD); (iv) Group D (QMix 2-in-1); and (v) Group E (aqueous extracts of *Azadirachta indica* and *Citrus limon*). After we prepared the canals with ProTaper Universal nickel-titanium (Ni-Ti) rotary files (Dentsply Sirona) and the respective irrigants, we split the teeth longitudinally to evaluate the amount of remnant smear layer in the coronal, middle, and apical thirds using SEM photomicrographs. We performed statistical analyses of the data using the Kruskal-Wallis and Mann-Whitney U tests, where the level of significance was set at 0.05.

Results: The SEM analysis of the coronal third showed mean values of 3.83 in Group A, 3.67 in Group B, 2.79 in Group C, 3.63 in Group D, and 4.00 in Group E. The SEM analysis of the middle third showed mean values of 4.00 in Group A, 3.88 in Group B, 3.75 in Group C, 3.50 in Group D, and 3.50 in Group E. The SEM analysis of the apical third showed mean values of 3.92 in Group A, 3.63 in Group B, 3.71 in Group C, 3.88 in Group D, and 3.17 in Group E. Therefore, we found that there were significant statistical differences between the groups when an overall comparison was done for the coronal, middle, and apical third, with a p-value of 0.001. On multiple comparisons across the different tooth-section thirds. Groups A and B showed statistically significant differences in the apical third (p-value=0.017). Groups A and C showed statistically significant differences in the coronal third and middle third (p-values=0.001 and 0.010, respectively). Groups A and D showed statistically significant differences in the middle third (p-value=0.001). Groups A and E showed statistically significant differences in all thirds (p-values=0.039, 0.001, and 0.001, respectively)

Conclusion: The conventional needle irrigation with MTAD showed the highest level of smear layer removal ability on the root canal surface, followed by QMix 2-in-1, the *Azadirachta indica* leaf and *Citrus limon* extract mixture, and the conventional endodontic regimen. Normal saline showed the lowest smear layer removal effect.

## Introduction

The primary objective of endodontic treatment is to eliminate microorganisms from the root canal system and prevent re-infection of the canal through proper obturation, before which the root canals should be thoroughly cleaned and shaped with mechanical instrumentation supplemented with irrigants and intracanal medicaments. An amorphous, irregular layer known as the smear layer has been shown to form on root canal walls following the mechanical preparation of the root canal system [1,2].

The smear layer is a substrate of a heterogeneous structure consisting of organic and inorganic particles, necrotic and viable tissue (e.g., remnants of odontoblastic processes and pulp tissue), and microorganisms (e.g., bacteria and fungi) and their byproducts [3,4]. It is recommended to remove the endodontic smear layer, as it is a loosely adherent structure that may act as both a potential avenue for leakage and bacterial contaminant passage between the obturating material and the canal wall [5] and a substrate for bacteria [6]. The smear layer also limits the intra-tubular penetration of disinfecting agents and endodontic sealers [1,7], and it can act as a barrier between the obturating material and the canal wall, compromising the formation of a satisfactory seal [8,9]. Finally, it can also affect the adhesiveness of luting cement in the case of the adhesion of fiber posts to the radicular dentin [10].

Although sodium hypochlorite (NaOCl) is a root canal irrigant that is widely used during endodontic treatment because of its antibacterial and tissue-dissolving properties [11], it cannot remove the smear layer efficiently [12,13]. Therefore, NaOCl is supplemented with ethylenediamine tetraacetic acid (EDTA) in smear layer removal [14,15].

To simplify the irrigation regimen, irrigants with antibacterial and smear layer removal abilities are combined and introduced commercially. For example, MTAD (mixture of tetracycline, acid, and detergent) (BioPure MTAD, Dentsply Sirona, Charlotte, North Carolina, United States) and QMix™ 2-in-1 (Dentsply Sirona), a mixture of chlorhexidine (CHX)-analog, triclosan, and EDTA, are newer irrigants with antimicrobial properties that aid in the removal of both the smear layer and organic tissue from the infected root canal system [16].

*Azadirachta indica* is a viable medicament against *Enterococcus faecalis* [17], whereas *Citrus limon* has been found to remove the smear layer and open the dentinal tubules [18]. This study compared the smear layer removal ability of a conventional endodontic irrigation regimen, MTAD, and QMix with that of aqueous extracts of *Azadirachta indica* and *Citrus limon*, by using SEM.

## Materials and methods

The study was conducted at Rajas Dental College and Hospital, Tirunelveli, India, and was approved by the Institutional Ethics Board at Rajas Dental College and Hospital (approval number: RDCH/IRB/2022/023). Forty straight, single-rooted, intact, human premolar teeth were collected for the study, which were extracted for orthodontic reasons. Teeth with curved roots, canal calcifications, and root caries were excluded from the study. After cleaning the selected teeth for debris and calculus, they were sectioned at the level of the cementoenamel junction with a diamond disc while maintaining a uniform root length of 16 mm and then stored in a 0.9% saline solution.

Grouping

**Figure 1:**
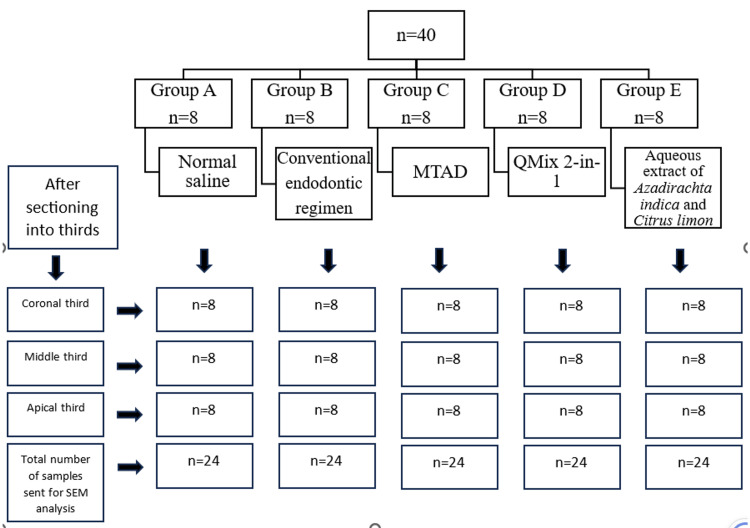
Sample grouping MTAD: mixture of tetracycline, citric acid, and detergent; SEM: scanning electron microscopy QMix™ 2-in-1, Dentsply Sirona, Charlotte, North Carolina, United States

We randomly divided the 40 samples into five groups (eight samples each): (i) Group A (normal saline), (ii) Group B (conventional endodontic regimen, 3%NaOCl+17%EDTA+2%CHX), (iii) Group C (MTAD), (iv) Group D (QMix 2-in-1), and (v) Group E (aqueous extract of *Azadirachta indica* and *Citrus limon*) (Figure 1).

We measured the working length of each sample with a #10 K-file (Mani, Inc., Tochigi, Japan), where the tip of the file was visible at the apical foramen. After obtaining the glide path with a #15 K-file, we prepared root canals of all the teeth using ProTaper Universal nickel-titanium (Ni-Ti) rotary files (Dentsply Sirona), up to a #F3 file, in accordance with the manufacturer’s instructions [19]. Throughout the instrumentation period, we used 2 mL of each of the respective irrigants for each group, except for the conventional regimen group, where 2 mL of 3% NaOCl was used.

The final irrigation delivery time and volume have been standardized for each group at 5 minutes and 5 mL, respectively. We irrigated the root canals of all the samples using a 30-gauge side-vented endodontic irrigation needle (TruNatomy Irrigation Needle, Dentsply Sirona).

In Group A, we carried out the irrigation with 0.9% saline. In Group B, we followed a conventional endodontic irrigation regimen, in which 2 mL of 3% of NaOCl solution (Prime Dental Products Pvt. Ltd., Thane, Maharashtra, India) was used, followed by 2 mL of 17% EDTA (Meta BioMed Co. Ltd., Republic of Korea), and then 1 mL of 2% chlorhexidine gluconate solution (Asep-RC; Anabond Stedman Pharma Research Pvt Ltd, Chennai, India) as a final rinse [20]. Samples in Group C were irrigated with MTAD solution, which was prepared fresh by mixing the MTAD powder and liquid as supplied by the manufacturer (Dentsply Sirona) and used according to the manufacturer’s instructions. Samples in Group D were irrigated with QMix 2-in-1 solution as per the manufacturer’s instructions (Dentsply Sirona). Samples in Group E were irrigated with a mixture of aqueous extracts of *Azadirachta indica* and *Citrus limon*. We prepared the aqueous extract of *Azadirachta indica* by macerating the extract with distilled water. The *Citrus limon* extract was obtained from the pulp of the citrus fruit. Finally, we rinsed the root canals with distilled water to remove any precipitate and dried them with paper points (ProTaper universal absorbent paper points-F3, Dentsply Sirona).

After irrigation, the teeth was sectioned by creating a vertical groove in the buccolingual direction with carborundum discs at low speed under continuous water irrigation. Then, each tooth was split vertically by applying slight pressure to an enamel chisel placed into the longitudinal groove. After longitudinal sectioning, we used one-half of the tooth with root canal space for cross-sectioning into coronal, middle, and apical thirds, while we discarded the other half of the longitudinally sectioned tooth with a faint canal (Figure 1).

We used SEM to assess the effectiveness of the final irrigants’ capacity to remove smear layers. Each sample was coded and mounted on an aluminum holder, sputter coated with gold, and then examined with SEM (Zeiss Evo 18; Carl Zeiss AG, Oberkochen, Baden-Württemberg, Germany) at 20 kV. We imaged the samples at a magnification of 2000X to assess the presence of a smear layer.

**Figure 2:**
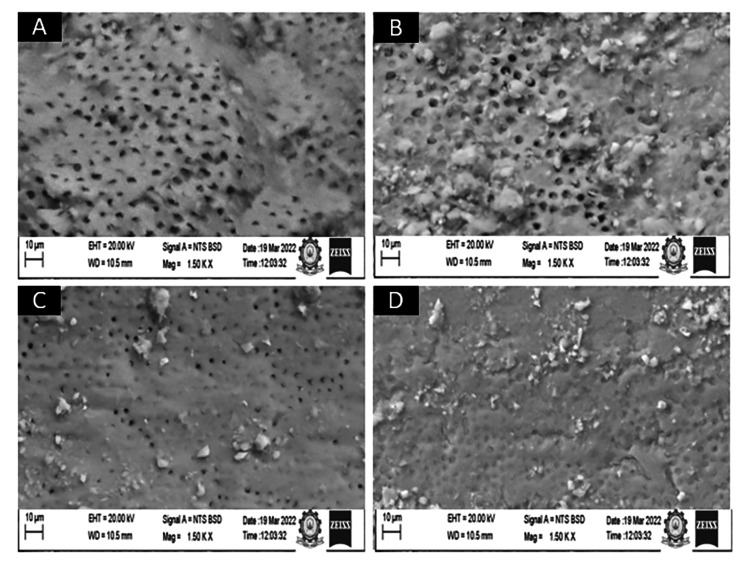
Scanning electron microscopy images using the Hulsmann scoring system (A) patent dentinal tubules; (B) patent dentinal tubules in >50% of the surfaces; (C) patent dentinal tubules in <50%of the surfaces; and (D) no patent dentinal tubules.

Two calibrated examiners, NDM and MM, assigned scores (1 to 4) to the SEM micrographs according to the criteria given by Hülsmann et al. [21], where a score of 1 meant the dentinal tubules were completely patent (Figure 2A), a score of 2 meant more than 50% of the dentinal tubules were patent (Figure 2B), a score of 3 meant less than 50% of the dentinal tubules were patent (Figure 2C), and a score of 4 meant nearly all of the dentinal tubules were occluded with smear layer (Figure 2D). Coincident scores between two examiners were assigned to each specimen. In the case of disagreement between the two examiners, the specimen was re-evaluated by a third examiner (NM). We tabulated the scores in a Microsoft Office Excel Sheet (Microsoft Corporation, Redmond, Washington, United States) and performed a statistical analysis.

Statistical analysis

We analyzed the data using the IBM SPSS Statistics for Windows, Version 21.0 (Relesed 2012; IBM Corp., Armonk, New York, United States). Statistical significance was set at a level of 5% (i.e., p<0.05 was considered statistically significant). On evaluation of the smear layer scores, the normality distribution test resulted in a p-value of <0.05. Intra-group comparisons were made using the Kruskal-Wallis test. Inter-group comparisons were made using the Mann-Whitney U test.

## Results

On multiple Hulsmann score comparisons, we found statistically significant differences between groups, with p-values of 0.026, 0.001, 0.001, 0.001, 0.010, and 0.050 for groups A and B, A and C, A and D, A and E, B and C, and C and D, respectively (Table 1).

**Table 1:** Multiple comparisons of the overall Hulsmann scores between groups. * Indicates statistical significance.

Group	Comparison group	Mean difference	p-value
Group A	Group B	−2.224	0.026*
	Group C	−4.527	0.001*
	Group D	−3.805	0.001*
	Group E	−3.521	0.001*
Group B	Group C	−2.574	0.010*
	Group D	−1.052	0.293
	Group E	−1.518	0.129
Group C	Group D	−1.956	0.050*
	Group E	−1.127	0.260
Group D	Group E	−0.906	0.365

The SEM analysis of the coronal third showed mean values and standard deviations of 3.83 and 0.381 in Group A, 3.67 and 0.702 in Group B, 2.79 and 1.021 in Group C, 3.63 and 0.576 in Group D, and 4.00 and 0.0 in Group E, respectively. In the inter-group analysis, the Kruskal-Wallis chi-squared value was 38.231 (Table 2). The SEM analysis of the middle third showed mean values and standard deviations of 4.00 and 0.0 in Group A, 3.88 and 0.338 in Group B, 3.75 and 0.442 in Group C, 3.50 and 0.722 in Group D, and 3.50 and 0.511 in Group E, respectively. In the inter-group analysis, the Kruskal-Wallis chi-squared value was 19.772 (Table 2). The SEM analysis of the apical third showed mean values and standard deviations of 3.92 and 0.282 in Group A, 3.63 and 0.495 in Group B, 3.71 and 0.550 in Group C, 3.88 and 0.338 in Group D, and 3.17 and 0.761 in Group E, respectively. In the inter-group analysis, the Kruskal-Wallis chi-squared value was 24.086. Therefore, we found that there were significant statistical differences between the groups when an overall comparison was done for the coronal, middle, and apical third, with a p-value of 0.001 (Table 2).

**Table 2:** Comparison of groups for each tooth section third. * Indicates statistical significance

		Mean	SD	Median	Minimum	Maximum	Kruskal–Wallis chi-squared	p-value
Coronal third	Group A	3.83	0.381	4	3	4	38.231	0.001*
	Group B	3.67	0.702	4	2	4		
	Group C	2.79	1.021	3	1	4		
	Group D	3.63	0.576	4	2	4		
	Group E	4.00	0.000	4	4	4		
Middle third	Group A	4.00	0.000	4	4	4	19.772	0.001*
	Group B	3.88	0.338	4	3	4		
	Group C	3.75	0.442	4	3	4		
	Group D	3.50	0.722	4	2	4		
	Group E	3.50	0.511	4	3	4		
Apical third	Group A	3.92	0.282	4	3	4	24.086	0.001*
	Group B	3.63	0.495	4	3	4		
	Group C	3.71	0.550	4	2	4		
	Group D	3.88	0.338	4	3	4		
	Group E	3.17	0.761	3	2	4		

We made multiple comparisons across the different tooth-section thirds. Groups A and B showed statistically significant differences in the apical third (p-value=0.017). Groups A and C showed statistically significant differences in the coronal third and middle third (p-values=0.001 and 0.010, respectively). Groups A and D showed statistically significant differences in the middle third (p-value=0.001). Groups A and E showed statistically significant differences in all thirds (p-values=0.039, 0.001, and 0.001, respectively) (Table 3). Groups B and C showed statistically significant differences in the coronal third (p-value=0.001). Groups B and D showed statistically significant differences in the middle and apical thirds (p-values=0.037 and 0.048, respectively). Groups B and E showed statistically significant differences in all thirds (p-values=0.020, 0.006, and 0.031, respectively) (Table 3). Groups C and D showed statistically significant differences in the coronal third (p-value=0.002). Groups C and E showed statistically significant differences in the coronal third and the apical third (p-values=0.001 and 0.007, respectively). Groups D and E showed statistically significant differences in the coronal third and the apical third (p-values=0.002 and 0.001, respectively) (Table 3).

**Table 3:** Multiple comparisons across tooth section thirds. * Indicates statistical significance.

Group	Comparison group	p-value		
		Coronal	Middle	Apical
Group A	Group B	0.585	0.077	0.017*
	Group C	0.001*	0.010*	0.118
	Group D	0.171	0.001*	0.640
	Group E	0.039*	0.001*	0.001*
Group B	Group C	0.001*	0.272	0.421
	Group D	0.483	0.037*	0.048*
	Group E	0.020*	0.006*	0.031*
Group C	Group D	0.002*	0.253	0.254
	Group E	0.001*	0.077	0.007*
Group D	Group E	0.002*	0.672	0.001*

## Discussion

Removal of the smear layer within the root canal system facilitates the diffusion of irrigants into the dentinal tubules, thereby improving the efficacy of the endodontic treatment and reducing the time required for disinfection of the canal [22]. Additionally, removal of the smear layer helps the obturating material and sealer better conform to the canal walls, which improves the apical seal. However, smear layer removal in the apical third of the root canal is critical because it addresses multiple problems, such as limited space, low permeability, and complex anatomical configuration [23].

For the elimination of the intra-radicular smear layer, several chemical irrigants, including EDTA, citric acid, and maleic acid, have been suggested [24]. According to different studies, using a combination of 2.5-5% NaOCl and 10-17% EDTA is effective in the removal of organic and inorganic debris [22,25]. Therefore, in the conventional regimen group, we also used 17% EDTA as a chelating agent along with NaOCl to remove the inorganic components of the smear layer during canal irrigation.

During the chemo-mechanical preparation of the root canal, irrigants such as EDTA remain in the canal only for a short period of time. The smear layer removal ability of EDTA depends on its concentration and contact time. Teixeira et al. estimated that canal irrigation with EDTA and NaOCl for one, three, and five minutes were equally effective in removing the smear layer from the canal walls of straight roots [26]. Therefore, in the present study, a contact time of five minutes was taken as the standard time for all the irrigants in all groups.

Owing to citric acid being one of the basic constituents in MTAD, researchers have evaluated its potential use in removing the endodontic smear layer. However, the findings were unfavorable [4,27]. Interestingly, *Azadirachta indica* and *Citrus limon* showed better smear layer removal ability than other irrigants at the middle and apical thirds. Such differences may be attributed to several factors [28]. First, the combination of *Azadirachta indica* and *Citrus limon* showed a synergistic ability to remove the smear layer, and it can be used as a beneficial adjunct in the final irrigation of root canals [27]. Second, even assuming the absence of any obstructing debris, injection of the irrigating solution into the coronal part of the canal may not penetrate to the apical part unless the needle is jammed into the canal [28]. Under normal circumstances, the fluid dynamics in a root canal lead to a “stagnation plane,” thereby hindering fluid penetration into the apical third. In real in vivo situations, intrusion of body fluid into the root canal system may interfere with the nature and effectiveness of the irrigants. We conclude that smear layer removal should be routinely practiced, particularly in cases of teeth with established periapical infections. [29].

Effective smear layer removal is a prerequisite for successful endodontic treatment. In the coronal third of the root canal, the smear layer removal ability of different root canal irrigating solutions was ranked as follows: MTAD > QMix 2-in-1 > conventional endodontic regimen > normal saline > mixture of *Azadirachta indica* and *Citrus limon*. In the middle third of the root canal, the smear layer removal ability of the different root canal irrigating solutions was ranked as follows: mixture of *Azadirachta indica* and *Citrus limon* > QMix 2-in-1 > MTAD > conventional endodontic regimen > normal saline. In the apical third of the root canal, the smear layer removal ability of the different root canal irrigating solutions was ranked as follows: mixture of *Azadirachta indica* and *Citrus limon* > conventional endodontic regimen > MTAD > QMix 2-in-1 > normal saline.

SEM and blind evaluations are most commonly used in various studies for sample observation and scoring methods, respectively. Although new technologies such as environmental SEM, atomic force microscopy, and co-site optical microscopy have been developed, they have their own shortcomings [30]. Therefore, further research is needed to develop and test new methodological approaches to assess smear layer removal.

This study has some potential limitations. The simulation of the root canal biofilm environment with a smear layer in in vitro studies may not be equivalent to the in vivo environment. To overcome this limitation, a randomized clinical trial should be emphasized in in vivo scenarios with long-term follow-up in a well-established center. We used the conventional method of needle irrigation in this study, rather than other modes of irrigation agitation systems, which could have been included to incorporate current trends in endodontic irrigation. Therefore, future studies should emphasize in vivo scenarios with long-term follow-up, where randomized clinical trials can be planned in well-established centers.

## Conclusions

Within the limitations of the present study, we conclude that, overall, conventional needle irrigation with MTAD showed the highest level of smear layer removal ability on the root canal surface, followed by QMix 2-in-1, a mixture of *Azadirachta indica* and *Citrus limon*, and NaOCl+EDTA+CHX. Normal saline showed the lowest smear layer removal effect. Based on the results of this study, it can be concluded that the mixture of herbal irrigants, namely, aqueous extracts of *Azadirachta indica* and *Citrus limon*, can also be used as an effective root canal irrigant for smear layer removal. Further studies with long-term follow-up should be carried out for in vivo scenarios to appropriately evaluate the efficacy of the mixture of herbal irrigants in cleaning the root canal.
